# The Efficacy of Administering Fruit-Derived Polyphenols to Improve Health Biomarkers, Exercise Performance and Related Physiological Responses

**DOI:** 10.3390/nu11102389

**Published:** 2019-10-07

**Authors:** Daniel S. Kashi, Akbar Shabir, Mariasole Da Boit, Stephen J. Bailey, Matthew F. Higgins

**Affiliations:** 1 School of Human Sciences, Derby University, Kedleston Road, Derby DE22 1GB, UK; D.Kashi@derby.ac.uk (D.S.K.); A.Shabir2@derby.ac.uk (A.S.); 2 School of Allied Health Sciences, De Montfort University, The Gateway, Leicester LE1 9BH, UK; mariasole.daboit@dmu.ac.uk; 3 School of Sport, Exercise and Health Sciences, Loughborough University, Epinal Way, Loughborough LE11 3TU, UK; S.Bailey2@lboro.ac.uk

**Keywords:** polyphenols, nitric oxide, antioxidant, health, phytochemicals, anthocyanins

## Abstract

Polyphenols are secondary metabolites involved in a myriad of critical processes in plants. Over recent decades, special attention has been paid to the anti-oxidative role of fruit-derived polyphenols in the human diet, with evidence supporting the contribution of polyphenols in the prevention of numerous non-communicable disease outcomes. However, due to the low concentration in biological fluids in vivo, the antioxidant properties of polyphenols seem to be related to an enhanced endogenous antioxidant capacity induced via signaling through the nuclear respiratory factor 2 pathway. Polyphenols also seem to possess anti-inflammatory and antioxidant properties and have been shown to enhance vascular function via nitric oxide mediated mechanisms. Consequently, there is rationale to support fruit-derived polyphenol supplementation to enhance exercise performance, possibly via improved muscle perfusion. Fruit-derived polyphenol supplementation in exercise studies have included a variety of fruits, e.g., New Zealand blackcurrant, pomegranate, and cherry, in the form of extracts (multicomponent or purified), juices and infusions to varying degrees of benefit. For example, research has yet to link the health-related benefits of black elderberry (*Sambucus nigra* L.) ingestion to exercise performance in spite of the purported health benefits associated with black elderberry provision in vitro and in vivo models, which has been attributed to their high antioxidant capacity and polyphenol content. This review summarizes the existing evidence supporting a beneficial effect of fruit-derived polyphenols on various biological processes and outlines the potential for black elderberry ingestion to improve nitric oxide production, exercise performance, and the associated physiological responses before-, during- and post-exercise.

## 1. Introduction

Polyphenols are described as secondary metabolites, and are involved in a wide range of critical processes in plants, including; growth, pigmentation, pollination, and resistance to pathogens and environmental stressors [1]. In recent decades, special attention has been paid to the anti-oxidative role of polyphenols in the human diet, with evidence supporting the contribution of polyphenols in the prevention of cardiovascular diseases [2], cancers [3], neurodegenerative diseases [4], and diabetes mellitus [5]. Total daily dietary intake of polyphenols can be as high as 1 g/day, which is ~10 to ~100 times higher than the intakes of other ‘phytochemicals’ and known dietary antioxidants, i.e., vitamin C, vitamin E, and carotenoids [6]. Structurally, polyphenols are characterized by two or more hydroxyl groups attached to one or more benzene rings. Variations in this chemical structure lead to the classification of polyphenols into one of four families, including; phenolic acids, flavonoids, stilbenes, and lignans. Due to both the low quantities and limited food sources, the contribution of stilbenes and lignans to total polyphenol intake is generally minor. Comparatively, phenolic acids and flavonoids, are considered to be the most common in the human diet, with main sources including; fruits, vegetables, fruit juices, tea, coffee, red wine, cereals, and chocolate [7]. 

The health promoting effects of diets rich in fruits and vegetables in reducing the risk of a number of diseases is well documented [8]. These beneficial effects have been attributed to the polyphenol contents of such diets [9] as evidenced by in vitro experimental models [10,11] and epidemiological studies associating dietary provision of fruit-derived polyphenols with reductions in the risk of non-communicable diseases [12,13,14]. However, the bioefficacy of polyphenols depends on their bioavailability. Indirect evidence of polyphenol absorption through the gut barrier is provided by the increase in the antioxidant capacity of the plasma after the consumption of polyphenol-rich foods, e.g., tea [15], red wine [16], blackcurrant, and apple juice [17]. More direct evidence on the bioavailability of a few phenolic compounds has been obtained by measuring their concentrations in plasma and urine after the ingestion of either pure compounds or of foodstuffs with a known content of the compound of interest. 

Initially, polyphenols are conjugated with sugar moieties within the plants (glycosides) and must be hydrolyzed to split the sugar group (glycone) from the polyphenol (aglycone) before absorption. This process occurs as a result of the action of lactase phloridzin hydrolase (LPH) in the brush border of the small intestine epithelial cells [18]. The aglycones can be absorbed into the circulation; however, they are subject to conjugation, resulting in the formation of sulfate, glucuronide, and/or methylated metabolites both within the aforementioned epithelial cells and the liver [19]. A large proportion of polyphenols are not absorbed within the small intestine and continue to the colon where they are acted upon by enzymes present within the microbiota to release aglycones that then undergo ring fission to produce bioavailable metabolites such as phenolic acids [19]. However, it is important to consider that there is a high degree of inter-individual variation in bioavailability dictated by differences in the gut microbiome and food processing of polyphenols [6]. Additionally, the understanding of polyphenol absorption and metabolism is somewhat complicated because of the many thousands of different polyphenol compounds present within plants, their interactions within the food matrix, and their conjugation to form a large number of different metabolites upon absorption [20]. Indeed, fruits and vegetables and fruit-derived polyphenol supplements contain a blend of polyphenols and thus the pharmacokinetics and metabolism after ingestion of whole foods or fruit-derived supplements are even more complex. Despite this, the influence of subclasses of polyphenol groups, like anthocyanins on clinical outcomes have been investigated. 

Anthocyanins, the water-soluble subclass of flavonoids, act as natural pigments causing purple, blue, red, and orange coloration to flowers, leaves, fruits, and vegetables. They are known to elicit vasoprotective properties such as antioxidant, anti-inflammatory, anti-atherogenic, and vasodilatory actions in vitro [21,22,23]. There is now a growing body of evidence also demonstrating improvements to vascular health following the intake of dietary anthocyanins (such as, cyanidin-3-*o*-glucoside (C3G) in vivo [24,25]. Indeed, anthocyanins may improve vascular permeability and vasodilation as evidence by reduced systolic (SBP) and diastolic blood pressure (DBP) [26,27,28,29]. Anthocyanin-induced vasodilation may be linked to enhanced nitric oxide (NO) production through a variety of mechanisms. Firstly, in vitro C3G has been shown to upregulate nitric oxide synthase (NOS) expression and activity, which catalyzes the five-electron oxidation of L-arginine to yield NO [30,31]. Secondly, (with specific reference to reductive hydroxyl groups on the phenol ring) anthocyanins might aid the reduction of nitrite [NO_2_] into NO in the stomach [32]. Lastly, anthocyanin provision has been shown to inhibit nicotinamide adenine dinucleotide phosphate (NADPH) oxidase, one of the key sources of superoxide production [33], and to induce nuclear respiratory factor 2 (Nrf2)signaling that elicits increased endogenous antioxidant capacity [34], both of which will reduce oxidative stress with the potential to increase NO bioavailability. Augmented NO production is known to have a variety of positive health benefits, including; lowering blood pressure, particularly SBP [35], preserving or improving endothelial function [36,37], increased cellular glucose uptake [38], and some evidence of improvement to exercise performance [39,40]. 

Black elderberry (BE; *Sambucus nigra* L.) is a rich source of a variety of polyphenols, (including flavonoids, e.g., anthocyanins C3G, cyanidin-3-*o*-sambubioside (C3S), hyperoside, isoquercetin, quercetin, rutoside, epicatechin-3-*o*-gallate), and vitamins (100 mg of BE equivalent to; ~20 mg vitamin C and folic acid, ~2 mg biotin and nicotinic acid amide) [41]. Research surrounding the pharmacological effects of BE has been extensive. For instance BE has been observed to; increase diuresis, a mediator of hypertension [41], enhance recovery against upper respiratory tract infections (URTI) [42], mediate neuropathic pain [43], alleviate pain associated with headaches [44], possess hypolipidemic properties, and protect against oxidative stress, with particular impact in mitigating the risks associated with cardiovascular diseases [37]. Much of the benefits associated with BE are attributed to an enhanced polyphenol/anthocyanin capacity, particularly the anthocyanins C3G and C3S [41]. Given the purported multifaceted health benefits associated with BE ingestion, and the increasing popularity of foods and supplements high in anthocyanins within sports and exercise nutrition [45], the current review highlights the potential and scope for BE to improve physiological responses during exercise and exercise performance. This is achieved by outlining the theoretical basis by which fruit-derived polyphenols might improve exercise performance, with particular emphasis on the potential efficacy of BE supplementation given its antioxidant capacity and potential to augment NO production.

## 2. Oxidative Stress and Exercise

During exercise, reactive oxygen species (ROS) are produced by skeletal muscle from a range of sources, including; phospholipase A2 (PLA2) and enzymatic sources such as NADPH oxidase and xanthine oxidase [46]. Paramagentic electron spin resonance experiments have confirmed that increases in exercise intensity are characterized by a concomitant increase in ROS generation [47,48]. ROS are important signaling molecules and have been implicated in contraction mediated increases in muscle glucose uptake and the control of skeletal muscle blood flow. For instance, hydrogen peroxide has been shown to cause vasodilation in exercising muscle [49]. It appears that under conditions of low oxidative stress and redox perturbation, i.e., during rest or low intensity exercise, ROS promote optimal vasodilation and hyperaemia in exercising muscle. In contrast, excessive development of ROS derived during intense physical exercise can impair blood flow and vasodilatory capacity. Moreover, excess ROS generation has been shown to impair calcium handling and sensitivity resulting in reduced contractile force development, thus impairing exercise performance [46,50]. 

During prolonged high intensity exercise where ROS generation exceeds the antioxidant capacity and results in disturbed redox balance, it is plausible that antioxidant supplementation may counteract fatigue and enhance performance via enhanced perfusion of the exercising muscle and better maintenance of excitation-contraction coupling. There is some evidence that the antioxidant capacity conferred by both acute and chronic supplementation with fruit-derived polyphenols, specifically anthocyanin subclass, is ergogenic. For instance, using a combined exercise and cellular model, acute ingestion of blackcurrant (240 mg anthocyanin) prior to 30 min rowing at 80% VO_2max_ attenuated exercise-induced ROS generating capability, i.e., plasma carbonyls (0.9 ± 0.1 versus 0.6 ± 0.1 nmol/mg protein, placebo versus blackcurrant) [51]. Such antioxidant capacity of blackcurrant may indirectly explain downwards shifts in lactate curves during incremental intensity cycling, greater total running distance, and increased preserved maximal sprint running observed following supplementation with New Zealand blackcurrant powder (NZBC) in trained cyclists (×1300 mg/day capsule for seven days = 105 mg of anthocyanin per serving) [52], untrained males (×1300 mg/day capsule for seven days = 105 mg of anthocyanin per serving) [53] and trained youth footballers males (×2300 mg/day capsule for seven days = 210 mg of anthocyanin per serving) [54], respectively. The effects of acute polyphenol supplementation on exercise performance have also included the ingestion of pomegranate. Positive findings have demonstrated that consumption of 1 g of pomegranate extract (polyphenol content not provided), consumed 30 min prior to exercise enhanced time to exhaustion whilst running at 90% and 100% of peak velocity achieved at V$\dot{O}$_2max_, by ~12% (388 ± 199 versus 346 ± 163 s) and ~8% (171 ± 66 versus 159 ± 62 s), respectively [53]. Additionally, a combined supplement of pomegranate, green tea, and grape extract (×2500 mg capsules = 290 mg polyphenolic bioactives) consumed acutely 1 h pre-exercise increased total power output (5%), maximal peak power output (4%), and average power output (5%) during repeated cycle Wingate tests in recreationally active individuals [54], without inducing a higher fatigue index in supplement versus placebo treatments (38 ± 13% versus 39 ± 10%, respectively) or greater post exercise heart rate (129 ± 15 beats.min^−1^ versus 132 ± 26 beats.min^−1^, respectively). However, trained cyclists [55] and moderately resistance-trained individuals [56] did not exhibit performance benefits from consumption of 1000 mg (×2500 mL solution = 1800 mg polyphenolic bioactives) of pomegranate extract. Furthermore, no ergogenic benefit was observed following more chronic pomegranate supplementation regimens in trained participants [57]. The lack of an ergogenic benefit following acute and chronic supplementation of pomegranate observed in trained individuals [55,56,57] might be owing to the improvements in cycling efficiency [58] and running economy [59] incurred by many years of specific endurance training. 

There is also limited evidence examining the influence of dietary enrichment with cherries, another fruit high in anthocyanins, on exercise performance. For example, in a single-blind design, Howatson et al. [60] examined the influence of supplementing two groups of recreational runners with either cherry juice (×28 oz bottle for five days = 600 mg phenolic compounds [40 mg anthocyanin] per serving) or placebo on marathon finishing time. No difference in marathon finish times was observed following five days of cherry juice (3:41:00 ± 0:26:01 hrs:mins:s) or placebo (4:15:48 ± 1:01:22 hrs:mins:s). However, the improvement of ~34 min for the cherry juice treatment (and Cohen’s d effect size of ~0.75 versus placebo) suggests an ergogenic potential of cherry juice treatment. Additionally, double blind provision with a powdered form of tart cherry skins (x1480 mg/day capsule for seven days = 991 mg of phenolic compounds [66 mg of anthocyanin] per serving) or placebo for seven days prior and on the day of a half marathon resulted in ~13% faster half marathon finishing times in the cherry group (1:43:00 ± 0:09:17 hrs:mins:s) compared to placebo (1:58:00 ± 0:09:43 hrs:mins:s) [61]. One study to date has examined the influence of acute ingestion of cherry juice on shorter duration exercise [62]. Despite observing no effect on time-to-exhaustion during severe intensity exercise following acute Montmorency cherry concentrate ingestion (polyphenol and anythocyanin content not reported), increases of ~10% in both peak power and total work during a 60 s all out sprint following the time to exhaustion protocol were observed [62]. 

## 3. Antioxidant Potential of Fruit-Derived Polyphenols: Mechanisms

The ergogenic effects of fruit-derived polyphenols appear to be associated with enhanced vascular function, and may result in improved muscle perfusion and enhanced oxygen extraction. For instance, greater increases in brachial artery diameter (diameter = 0.42 ± 0.07 cm versus 0.39 ± 0.07 cm) and blood flow (flow = 40.6 ± 24.8 mL·min^−1^ versus 29.6 ± 24.9 mL·min^−1^) paralleled the aforementioned ergogenic effects on performance following pomegranate compared with placebo supplementation [53]. Additionally, Cook et al. [63] found that seven days of supplementation with 600 mg of blackcurrant powder resulted in 6.9%–8.2% increases in femoral artery diameter measured at different stages during two min of sustained submaximal maximum voluntary contraction (MVC) of the knee extensors (30% MVC). Accompanying these effects were 7%–12% reductions in SBP, 7%–9% lower DBP and 16%–25% lower total peripheral resistance at various time points during the 120 s MVC protocol, estimated via beat-beat singer blood pressure analysis. However, it is important to note that participants ingested twice the daily dose of blackcurrant powder ingested compared to the aforementioned exercise performance studies [51,64]. These beneficial effects on vascular function were classically attributed to the potential of polyphenols to scavenge free radicals by B ring hydroxyl groups and conjugated double bonds. Resulting effects are a reduced exposure to/or an increased capacity to degrade ROS and reactive nitrogen species (RNS), as evidenced by reduced markers of oxidative stress or increased antioxidant enzyme activity. However, there is now growing evidence that polyphenols and the anthocyanin subclass are able to upregulate endogenous antioxidant capacity via signaling pathways, particularly the nuclear factor erythroid factor 2-related factor 2 (Nrf2). Nrf2 is a master regulator and transcription factor, involved in regulating gene expression of antioxidant proteins. Keap1 is a cysteine-rich protein that represses Nrf2 signaling by serving as a bridge between Nrf2 and ubiquitination ligase cullin-3, which is required for the ubiquination of the protein and subsequent proteasomal degradation [65]. Oxidative stressors induce covalent modification of Keap1 cysteine residues and therefore inhibit ubiquitination dependent degradation and increase nuclear accumulation of Nrf2, resulting in increased synthesis of downstream endogenous antioxidants [66]. It is important to note that dietary polyphenols are not present in sufficient quantity in vivo to contribute directly to antioxidant function as radical scavengers. However, polyphenols are instead converted to electrophilic quinones and hydroquinones upon exposure to ROS, which are then able to interact with Keap1 and activate Nrf2 [67].

Upregulation of NfR2 has been associated with increased endurance exercise performance in mice, and though similar responses are possible in humans, such observations are yet to have been made [68]. Given the high anthocyanin content of BE it is conceivable that anti-oxidative effects and potential beneficial effects on exercise performance might be seen following the provision of BE. Black elderberry-derived polyphenols may provide particular potent antioxidant properties by increasing the volume of circulating antioxidant molecules [41], leading to free radical neutralization, and reductions in oxidative stress [41,69,70,71,72,73]. Much of the anti-oxidative capacity associated with BE may be attributed to the antioxidant promoting anthocyanins, C3G, and C3S. In support, eight healthy, non-smoking participants were assessed in two separate studies (two months apart) for total renal excretion of anthocyanins (study one), and changes in plasma anti-oxidative capacity (study two). In study one, participants ingested 200 mL (361 mg anthocyanins), 300 mL (541 mg anthocyanins), or 400 mL (722 mg anthocyanins) BE juice over a 7 h period, with each trial separated by a two-week washout period. Maximum anthocyanin excretion rates, as measured in urine, were observed at 1.5 h for 200 mL BE, 0.5 h for 300 mL BE, and 1 h for 400 mL BE juice, respectively. In general, total anthocyanin excretion did not exceed 0.05% at any dosage suggesting the majority of anthocyanins associated with BE juice dosages between 200–400 mL may have been preserved or metabolized in vivo, thus augmenting overall anti-oxidant capacity. For study two, participants ingested a one-off acute bolus of either 400 mL BE (2240 mg phenolics and 710 mg anthocyanins) or water. Baseline plasma trolox equivalent antioxidant capacity (TEAC), total radical-trapping antioxidant parameter (TRAP), and total polyphenol levels increased by 17%, 28% and 33% at 1, 2 and 4 h post-BE ingestion, respectively, with peak values reported after 1 h. 

BE-derived antioxidants have also been reported to improve endothelial function by mediating the expression of various cell adhesion molecules (CAM) and by regulating the cell redox status [70,74]. Cell adhesion molecules promote a pro-oxidant environment via overproduction of macrophages, which induce significant concentrations of ROS [69]. Black elderberries’ capability to reduce CAM activation is associated with improved cellular function via down regulation of ROS and nuclear factor-κB (NFκB) transcription factor [75]. NFκB has been observed to promote activation of inflammatory and CAM genes thus the downregulation of CAM, ROS, and NFκB may therefore improve endothelial function via anti-inflammatory responses [69]. Hepatic-ischemia reperfusion induced damage has also been observed to increase the activation of pro-inflammatory cytokines, tumour necrosis factor-α (TNF-α), and interleukin-1 β (IL-1β), known to induce CAM expression [74,76]. The BE derived anthocyanin C3G has been observed to enhance the resistance of endothelial cells to ROS, and subsequently protect against hepatic-ischemia reperfusion induced damage [77]. 

## 4. Nitric Oxide Metabolism: Mechanisms

As highlighted, there is potential for an ergogenic effect of fruit-derived polyphenols, including BE, with these effects likely underpinned by vascular and antioxidant mechanisms. An increase in anti-oxidative capacity from fruits high in polyphenols may improve vascular function by direct and indirect activation of the potent vasodilator, NO. Nitric oxide is a gaseous physiological signaling molecule, first recognized as a vasodilator capable of relaxing the vascular endothelium in cyclic guanosine monophosphate (cGMP)-dependent [78] and independent manners [79]. However, NO is also known to influence an array of physiological processes, including; mitochondrial respiration, neurotransmission, and skeletal muscle glucose uptake [80]. Nitric oxide is synthesized at several locations in the body by ubiquitously expressed NOS enzymes; endothelial (eNOS), neuronal (nNOS), and inducible (iNOS) isoforms. These enzymes catalyze the complex five electron oxidation of L-arginine to yield NO and L-citrulline in a reaction requiring oxygen (O_2_), nicotinamide adenine dinucleotide phosphate (NADPH), flavin adenine dinucleotide (FAD), flavin monocucleotide (FMN), tetrahydrobiopterin (BH4), haem, and calmodulin as substrates/co-factors. The production of NO is compromised by a reduced bioavailability of any of previously listed essential components. An additional O_2_ independent NO generating pathway has also been identified whereby NO is formed by a one electron reduction of nitrite [NO_2_^−^] [81].

Nitric oxide has received much interest in exercise physiology, and supplements that can increase NO synthesis have clear potential as an ergogenic aid [82]. This is largely based on the evidence that NO has been shown to be an important modulator of blood flow, mitochondrial respiration, and contractile function during physical exercise [83]. [NO_2_^−^] is the main oxidation product of NO in plasma and sensitively reflects acute and chronic changes in endothelial NO synthase (eNOS) activity in healthy volunteers under fasting conditions [84]. Several studies have also reported that plasma [NO_2_^−^] is positively associated with exercise capacity in humans [84,85,86,87]. Alternatively, [NO_2_^−^] infusion has been reported to cause vasodilation, which supports the idea that [NO_2_^−^] may act as NO donor in certain conditions [88]. It has been assumed that NO is involved in endothelium-mediated vasodilation and that this is one of the regulatory mechanisms by which substrate supply to working muscles is increased, thus allowing prolonged exercise [88]. As endothelium-derived NO is necessary to maintain an adequate vascular response to the increased blood flow demands during exercise, endothelial function is believed to be important for vasodilation induced by exercise. The fall in peripheral vascular resistance is a major factor for the increase in cardiac output during exercise, and is also mediated by endothelium-induced vasodilation [89]. Anthocyanin-induced vasodilation may be linked to enhanced NO production through a variety of mechanisms. Firstly, in vitro models, C3G has been shown to upregulate nitric oxide synthase (NOS) expression and activity [30,31]. An increased muscle blood flow may increase the oxidative energy contribution over the initial stages of exercise and in turn reduce the progressive increase in O_2_ uptake ($\dot{V}$O_2_) as high intensity exercise is continued facilitating improved exercise performance [90]. 

There is evidence to suggest that BE may propagate NO production in the stomach via the reduction of [NO_2_^−^], leading to the formation of NO intermediates such as *S*-nitrosothiol and in turn enhance NO and vascular function directly [91]. Therefore, oral BE supplementation might have the potential to improve aspects of $\dot{V}$O_2_ kinetics during exercise by enhancing endothelial function with the potential to improve exercise performance. This may, in part, be explained by the ability of BE polyphenols to promote NO production by stimulating phosphorylation of eNOS [80,92]. A recent cell culture study [93] demonstrated BE extract augmented NO production by ~70% compared to blackcurrant, ~60% compared to bilberry, and slightly improved NO augmentation (>3%) versus a lipopolysaccharide control. Further, C3G, an anthocyanin found in abundance in BE, has been shown to increase eNOS expression, which resulted in an enhancement of NO release [30]. Taken together, although unexplored in the context of BE supplementation, augmentation of NO might improve skeletal muscle perfusion and metabolism leading to enhanced exercise performance. 

## 5. Considerations for Future Research

Although there are no current studies investigating the influence of BE on exercise performance, the potential of any ergogenic effect will likely depend on several inter-related factors. For instance, and with reference to the influence of other fruits high in anthocyanins on exercise performance, varied findings are potentially owing to differences in intensity and duration of exercise protocols. In the studies by Howatson et al. [60] and Levers et al. [61], where equivocal and modest support for the ergogenic potential of fruit-derived anthocyanin supplementation was shown, the exercise durations were long at a moderate-intensity (~110 min–5 h). By comparison, consistently positive findings have been shown for a variety of fruit-derived anthocyanin supplements utilizing more intense and intermittent exercise protocols, with intense bouts lasting between ~10 s to 15 min [51,54,62,64]. The vasodilatory capability of anthocyanins and subsequent effects on cardiac output [63] may benefit exercise characterized by an imbalance of perfusion to support metabolism and cause a decrease in intramuscular oxygen partial pressure [94] and acidic conditions [95], both of which are apparent during high intensity exercise. 

Previous studies have also used a variety of supplementation regimens. The studies by Cook et al. [96], Murphy et al. [97], Perkins et al. [51], and Willems et al. [64] observed blackcurrant extract taken for six days before and on the morning of the seventh day, 2 h before performance testing. It is possible in these studies that performance benefits may have occurred as a result of the last dose, or as a product of bio-accumulation of anthocyanin metabolites incurred by the previous six days of supplementation. To date, no studies have investigated the possibility of bio-accumulation of BE metabolites and diverse tissue distribution of anthocyanins in humans following multiple days intake of BE. However, one study has observed that following an acute intake of 250 mL of blueberry juice, metabolites of anthocyanins are still present in urine five days following no further intake of anthocyanins [98]. Therefore, bio-accumulation of metabolites by anthocyanin intake is possible, and may mediate the aforementioned exercise performance benefits [51,64,96,97]. 

It is known that anthocyanins, anthocyanin metabolites, and other polyphenols can act synergistically [99]; therefore, the implementation of dietary control is an important consideration. The use of ‘washout’ periods allows the study design to control for potential interactions. In addition by removing polyphenols from the diet, it is possible that the potential for change is altered and it could be argued that the ergogenic effects are only of interest when they can be observed imposed on top of normal dietary intake, as in the design used by Levers et al. [61]. 

BE provision may have an ergogenic effect underpinned by vascular and antioxidant mechanisms that are characterized by an alteration in blood flow; similar to those observed in other foods high in anthocyanins [53,63]. However, the effects of regular polyphenol intake are an important consideration, given that increased antioxidant activity may potentially dampen training adaptations [100,101]. ROS produced during exercise have a requisite physiological role, i.e., they behave as signals to modulate adaptations to exercise [102,103]. Paramount among these adaptations is the change in the rate of mitochondrial biogenesis, which is dependent on oxidant-induced activation of factors such as peroxisome proliferator-activated receptor gamma coactivator 1α (PGC-1 α), nuclear respiratory factor 1 (NRF-1), and mitochondrial transcription factor A (mTFA) [101]. However, it should be acknowledged that these concerns are mostly raised against the promotion of long-term dosing of single antioxidants [104]. For instance, a combination of high intensity exercise and Ubiquinone-10 supplementation (120 mg for 20 days), increased cell damage, as evidenced by increased plasma creatine kinase, attributed to increased free radical production [105]. Currently it is not known whether the antioxidant potential of foods diverse in anthocyanins, and specifically BE, have comparable effects. However, there is some evidence of potentially positive benefits of regular anthocyanin intake. Indeed, oral ingestion of anthocyanins in mice has been shown to enhance exercise performance by activating lactate metabolism through skeletal muscle PGC-1α upregulation [106]. Extrapolation of these findings to human muscle during a period of physical training could provide positive benefits of regular anthocyanin intake on training adaptations. However, this is speculative and therefore further research on the potential combined effects of anthocyanin intake and physical training on biological adaptations is required. Study designs that incorporate the use of muscle biopsies may also allow the direct assessment of signaling pathways and changes in antioxidant enzyme activity in muscle after polyphenol supplementation. 

## 6. Conclusions

The consumption of extracts, juices, and infusions of certain fruits have been observed to ameliorate various biomarkers of health, possibly attributable to the high and diverse polyphenol content and antioxidant capacity of such fruit products. Based on previous literature demonstrating the ergogenic potential of other fruit-derived polyphenols, and in particular the anthocyanin subclass, we propose that the high polyphenol content and/or antioxidant capacity of BE (or its derivatives) may enhance exercise performance. This potential ergogenic effect of BE supplementation might arise as a result of lower oxidative stress and augmented NO production, and subsequent improvements in vascular function and skeletal muscle perfusion and metabolism. On the basis of these potential mechanisms, the present review highlights the need for future empirical research to analyze the possible ergogenic effects of BE ingestion on exercise performance. 
